# Supplementary material for: Saccharomyces boulardii (CNCM I-745) improves intestinal damage in sepsis by remodeling intestinal flora structure
Source: Front Cell Infect Microbiol. 2025 Jul 31;15:1602792. doi: 10.3389/fcimb.2025.1602792 (PMC12350329; doi:10.3389/fcimb.2025.1602792)
Supplement: Supplementary file 1 [file DataSheet1.zip › summary/summary/8_Advanced_analyse/2_PICRUSt2_result/KEGG/Group.DW0_vs_CW0_vs_BW0_vs_AW0/PICRUSt2_KEGG_level2_Group.DW0_vs_CW0_vs_BW0_vs_AW0_diff.pdf]

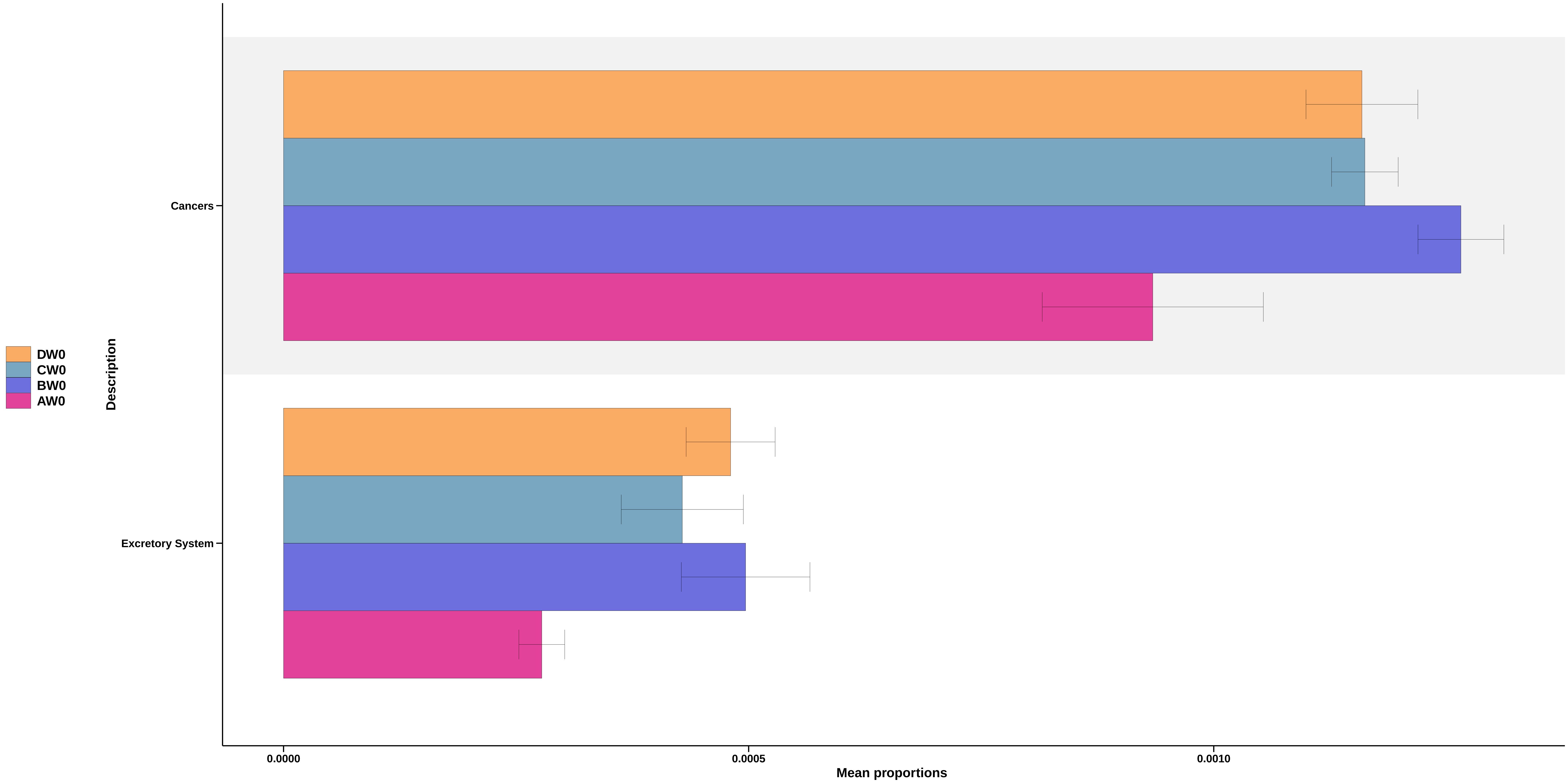
DW0
CW0
BW0
AW0
Description
Cancers
Excretory System
0.0000
0.0005
0.0010
Mean proportions
